# Metagenomic study in a predominantly adult cohort with hepatitis of unknown etiology

**DOI:** 10.1016/j.bsheal.2026.09.003

**Published:** 2026-09-18

**Authors:** Xinxin Li, Yuanjiao Gao, Ziyu Zhang, Yaqin Zhang, Weihua Cao, Wen Deng, Shiyu Wang, Xin Wei, Zixuan Gao, Linmei Yao, Shuojie Wang, Yao Xie, Minghui Li

**Affiliations:** a Department of Hepatology Division 2, Beijing Ditan Hospital, Capital Medical University, Beijing 100015, China; b Department of Hepatology Division 2, Peking University Ditan Teaching Hospital, Beijing 100015, China; c Hepatitis B Virus Infection, Clinical Cure and Immunology Joint Laboratory for Clinical Medicine, Capital Medical University, Beijing 100069, China

**Keywords:** Hepatitis of unknown etiology, Hepatitis of unknown origin (HUO), Metagenomics, Viruses, Goose circovirus (GoCV), Influenza A virus

## Abstract

Hepatitis of unknown origin (HUO) remains a diagnostic challenge, with metagenomic studies underexplored in adult cohorts. This study characterized the plasma and fecal virome in a predominantly adult HUO cohort (48 adults and 1 adolescent). A total of 59 samples (44 plasma, 15 fecal) from 49 patients were subjected to ultra-multiplex targeted ribonucleic acid (RNA) sequencing (139 RNA viruses) and unbiased metagenomic sequencing. Targeted sequencing detected HCV in one plasma sample, confirmed by follow-up reverse transcription-polymerase chain reaction (RT-PCR) and seroconversion, leading to case reclassification. Metagenomic sequencing identified HBV sequences in another patient, along with sporadic goose circovirus (GoCV) and an unconfirmed low-level influenza A-like signal. Viral community analysis revealed distinct niche-specific profiles between plasma and feces. Overall, this exploratory study failed to identify a common pathogenic agent, underscoring the etiological heterogeneity of HUO. The detection of known hepatotropic viruses highlights the diagnostic value of metagenomic sequencing in complementing conventional serology and reclassifying some HUO cases. Larger controlled studies are warranted.

## Highlights

**Scientific questionss:** Whether metagenomic and targeted sequencing approaches can identify known or novel pathogenic agents in predominantly adult patients with hepatitis of unknown origin (HUO), and whether plasma and fecal viromes exhibit distinct compositional profiles in this population.
**Evidence before this study:** Metagenomic sequencing has been extensively applied to pediatric hepatitis outbreaks, but its systematic application to predominantly adult HUO cohorts remains limited, leaving the etiological landscape, viral community structures, and compartment-specific virome characteristics largely unexplored in this age group.
**New findings:** No single virus was consistently enriched across the cohort; targeted sequencing identified hepatitis C virus (HCV) in one case, while metagenomic sequencing detected hepatitis B virus (HBV), goose circovirus (GoCV), and an unconfirmed low-level influenza A-like signal in one plasma sample (RPM < 2 threshold). Viral communities showed marked divergence between plasma and fecal compartments, with minimal overlap.
**Significance of the study:** This study underscores the etiological heterogeneity of HUO, highlights the complementary value of metagenomic sequencing in reclassifying cases initially negative by conventional serology, and provides the first comparative characterization of plasma and fecal viromes in a predominantly adult HUO cohort.

## 1 Introduction

Hepatitis, defined as liver inflammation, represents a significant global health burden with established etiologies including hepatotropic viruses, alcohol, drug toxicity, autoimmune responses, and metabolic disorders [1]. However, conventional diagnostic approaches fail to identify causative agents in a substantial proportion of clinical cases, now classified as “hepatitis of unknown origin” (HUO) or “non-A-E hepatitis” [2]. As a persistent enigma in clinical hepatology, HUO encompasses liver inflammatory diseases that remain undiagnosed after standard serological, biochemical, and imaging evaluations. Epidemiologically heterogeneous, these cases range from sporadic community-acquired episodes to spatiotemporal clusters of acute hepatitis, posing not only diagnostic and therapeutic challenges but also emerging as public health priorities due to potential transmissibility and unpredictable severe outcome [3]. The clinical burden of HUO extends beyond pediatric populations, with adults accounting for a significant and often overlooked proportion of cases. Recent epidemiological data indicate that chronic progressive cases in adults constitute a critical disease burden, with insidious progression to liver fibrosis, cirrhosis, and even liver failure [4], [5], [6]. Cases in adults frequently present with more protracted clinical courses, non-specific symptoms, and delayed diagnosis, complicating clinical management and compromising long-term prognoses [7]. The concept of idiopathic hepatitis is historically rooted-the systematic investigation of “non-A-non-B hepatitis” decades ago led to the landmark discovery of hepatitis C virus, exemplifying how etiological exploration of unexplained hepatitis can yield transformative virological breakthroughs. However, despite advances in diagnostic technologies, a substantial diagnostic gap persists, with up to 10%–20% of adult hepatitis cases remaining etiologically unresolved in routine clinical practice [8]. This persistent uncertainty not only complicates evidence-based therapeutic decisions but also hampers public health surveillance and outbreak preparedness.

In recent years, metagenomic next-generation sequencing (mNGS) has emerged as a powerful unbiased tool for pathogen discovery, enabling simultaneous detection of known and novel microorganisms directly from clinical specimens without prior hypotheses [9]. This technology has proven particularly valuable in investigating unexplained infectious diseases, including encephalitis, pneumonia, and sepsis, by identifying rare or unexpected pathogens [10]. However, its systematic application to predominantly adult HUO cohorts remains limited, with most metagenomic investigations concentrated on pediatric hepatitis outbreaks.

Following the 2022 multinational cluster of severe acute hepatitis in children, intensive research efforts focused on pediatric populations, generating hypotheses involving adenovirus, adeno-associated virus type 2, and immune-mediated mechanisms [11], [12], [13]. Nevertheless, these studies have not established a definitive causal pathogen, and more critically, they have created a significant knowledge gap regarding etiological investigations in HUO cases outside the pediatric age group. The pathogen spectrum, host immune responses, and environmental interactions in these patients may differ substantially from those in children, warranting dedicated investigation. The predominant focus on pediatric populations may obscure important age-specific pathogenic mechanisms and delay diagnostic advancements applicable to the broader HUO-affected adult population.

Based on the aforementioned background, this study aims to conduct an in-depth exploration of a patient cohort with HUO (predominantly adults) utilizing metagenomics as a powerful unbiased approach. We collected plasma and fecal specimens from HUO patients meeting strict inclusion criteria, complemented with comprehensive clinical data including liver biochemistry and hematological profiles. The exploratory objective of this study is to characterize the plasma and fecal virome in this patient population and to investigate whether any known or novel pathogens can be identified. The findings are expected to provide new microbial insights for etiological exploration, deepen our understanding of HUO pathophysiology, and potentially offer diagnostic refinements for future clinical practice, thereby carrying significant clinical value and scientific importance.

## 2 Materials and methods

### 2.1 Study population

This study enrolled 49 patients (48 adults and one adolescent aged 16 years) diagnosed with HUO at Beijing Ditan Hospital, Capital Medical University, between June 2024 and December 2024. The diagnosis of HUO required meeting all of the following criteria: (1) clinical and biochemical evidence of hepatitis [alanine aminotransferase (ALT) or ‌aspartate aminotransferase (‌AST) > 1.5 times the upper limit of normal (ULN) ], the ULN for ALT and AST in our center are 40 U/L and 35 U/L, respectively; (2) negative serological markers for acute or chronic viral hepatitis, defined as: immunoglobulin M (IgM) antibodies against HAV and HEV; hepatitis B surface antigen (HBsAg) and IgM antibodies against hepatitis B core antigen (anti-HBc IgM) for HBV; antibodies against HCV (anti-HCV); and total antibodies against HDV (anti-HDV). All serological tests were performed using commercially available chemiluminescent immunoassays (CLIA), and results were interpreted as negative based on the manufacturer’s recommended cut-off values. In addition, HCV RNA was tested by quantitative reverse-transcription PCR (RT-qPCR) with a lower limit of detection of 15 IU/mL, and HBV DNA was tested by quantitative PCR with a lower limit of detection of 20 IU/mL, both of which were required to be negative or below the detection limit for enrollment; (3) exclusion of alcoholic liver disease (alcohol consumption < 30 g/day for men; <20 g/day for women); (4) exclusion of drug-induced liver injury by detailed medication history review; (5) exclusion of autoimmune hepatitis (negative autoantibodies and normal immunoglobulins); (6) exclusion of metabolic liver diseases (ceruloplasmin, iron studies within normal limits); (7) exclusion of biliary obstruction by imaging. We adopted the existing liver function test results from the time of patient consultation or referral as diagnostic criteria, specifically ALT or AST levels > 1.5 times the ULN. Although some patients’ levels may have declined from their peak by the time of enrollment, their medical records clearly documented prior results meeting these abnormal criteria. Combined with persistent clinical symptoms, this still confirms a diagnosis of active hepatitis. All 49 enrolled patients fulfilled all seven exclusion criteria based on the standard clinical workup available at the time of presentation. It is important to emphasize that the diagnosis of HUO was based on this initial clinical evaluation; the subsequent identification of viral sequences through metagenomic sequencing, as described in the Results, represents new findings that may lead to reclassification of individual cases.

Demographic characteristics (age, sex, occupation, residence), epidemiological records (recent travel history, exposure history, vaccination status), clinical manifestations (symptoms, physical signs, disease progression), laboratory findings (liver function indices, hematological profiles), and imaging results were comprehensively documented.

### 2.2 Sample collection

Sample collection strictly adhered to international biobanking best practice guidelines. The specimens comprised 44 EDTA-anticoagulated plasma samples, which were centrifuged at 3,000 rpm within 2 h of collection and subsequently cryopreserved at − 80 °C, along with 15 sterile fecal samples obtained from 14 patients (including paired acute- and recovery-phase specimens from one patient). Additional supplementary specimens—including serum, blood clots, and peripheral blood mononuclear cells—were also collected. Among the 49 enrolled patients, 43 provided at least one plasma sample (33 contributed a single blood sample, and 9 provided matched plasma–fecal pairs), with one patient contributing two time-point plasma samples, yielding a total of 44 plasma specimens (Table S1). Fecal samples were collected from 14 patients, resulting in 15 specimens (including one paired acute-recovery set). For metagenomic sequencing, 59 optimal specimens (44 plasma and 15 fecal) were prioritized based on the following criteria: (1) for symptomatic patients, collection within 72 h of symptom onset; (2) for asymptomatic patients, collection within 72 h of the first detection of abnormal liver tests or diagnosis; (3) preservation integrity, defined as no prior freeze–thaw cycles; (4) completeness of associated clinical metadata. All specimens were anonymized through numerical coding. The study protocol involved standardized sample processing at Beijing Ditan Hospital, where serum samples were collected, processed, and stored at −80 °C. Subsequent virological analyses were conducted at the Chinese Center for Disease Control and Prevention (China CDC), following a rigorous workflow: nucleic acid extraction and preprocessing, library preparation and high-throughput sequencing. The generated sequencing data underwent comprehensive metagenomic analysis using Metagenomic Analysis Pipeline for Virome (MapV) [14], an in-house developed bioinformatics platform at China CDC capable of simultaneous detection of known and novel viral pathogens with high sensitivity.

### 2.3 Study design and experimental workflow

This investigation employed an integrated multi-omics strategy to systematically analyze 59 HUO specimens (44 plasma samples from 43 patients, and 15 fecal samples from 14 patients) using two complementary high-throughput sequencing approaches for etiologic profiling. The protocols for nucleic acid extraction are summarized in Table S2.

### 2.4 Nucleic acid extraction

All nucleic acid extractions were performed strictly according to the manufacturers' protocols. For both plasma and fecal samples, total nucleic acids (including both DNA and RNA) were extracted using magnetic bead-based kits. Briefly, total nucleic acids were extracted from plasma samples using a magnetic bead-based total nucleic acid extraction kit (Nucleic Acid Extraction Rapid Kit, Magnetic Bead Method, Bioperfectus, Jiangsu, China). For fecal samples, total nucleic acids were extracted using a magnetic bead-based fecal pathogen nucleic acid extraction kit (Ex-DNA/RNA Fecal Pathogen Nucleic Acid Extraction Kit, Bioperfectus, Jiangsu, China). Extracted total RNA was reverse-transcribed into complementary deoxyribonucleic acid (cDNA) using the SuperScript IV First-Strand Synthesis System (Invitrogen). Subsequently, both DNA and cDNA were randomly amplified using multiple displacement amplification (MDA) technology (Qiagen). Amplified products were purified using DNA purification and size selection beads, followed by washing with 75% ethanol. Purified nucleic acids were eluted in 50 μL of ultrapure water, and quantified using a Qubit fluorometer for quality control.

### 2.5 Library preparation and sequencing strategies

#### 2.5.1 Hyperplex targeted RNA sequencing

A multiplex polymerase chain reaction (PCR) assay using a custom primer pool covering 139 RNA viral species across 20 viral families (including 10 prioritized viruses from the WHO X-disease list) was performed on cDNA to construct targeted sequencing libraries [15], [16]. This panel included all pathogens from the 2023 National List of Human Pathogenic Microorganisms: Category 1 pathogens (15 species), Category 2 pathogens (27 species), and Category 3 pathogens (97 species). Libraries were sequenced on an Illumina MiSeq platform (SE75 mode), generating an average of 0.75 million reads per sample.

Due to a pending patent application and commercial confidentiality agreements with the collaborating company, the detailed primer and probe sequences are not publicly disclosed at this stage. However, the complete list of the 139 targeted viral species, along with their corresponding targeted bp genomic regions, is provided in Table S3 to ensure methodological transparency and allow readers to assess the scope of the screening panel.

#### 2.5.2 Metagenomic sequencing

Purified DNA and cDNA were pooled for library preparation, which was conducted by Novogene Co., Ltd. (Beijing, China). Libraries underwent deep sequencing on an Illumina NovaSeq 6000 platform (PE150 mode), yielding an average of 24.1 million paired-end reads [7.24 gigabase pairs (Gbp)] per sample.

### 2.6 Bioinformatics analysis pipeline

A systematic bioinformatics pipeline was employed for data analysis. The generated sequencing data underwent comprehensive metagenomic analysis using MapV (Metagenomic Analysis Pipeline for Virome) [17], an in-house developed bioinformatics platform at China CDC capable of simultaneous detection of known and novel viral pathogens with high sensitivity.

MapV is an integrative pipeline that systematically orchestrates a series of standard bioinformatics tools into a unified workflow. The pipeline architecture comprises several sequential modules. The first module is quality control and preprocessing, in which raw sequencing data were first assessed for quality using FastQC. Adapters and low-quality bases were trimmed using Trimmomatic to obtain high-quality clean reads for downstream analysis. The second module is host read depletion, in which quality‑controlled reads were aligned to the human reference genome GRCh38 using Bowtie2 to remove host‑derived sequences, yielding high‑quality non‑host sequences for downstream analysis. Host‑matching reads were subsequently filtered out. The third module is a dual‑track viral identification strategy. The first track consists of alignment‑based screening, in which non‑host reads were aligned against a comprehensive National Center for Biotechnology Information (NCBI) RefSeq pathogen database (updated quarterly) using Bowtie2, enabling rapid identification of known viral sequences. The second track is assembly‑based novel virus discovery: to capture sequences not fully covered by reference genomes, de novo assembly was performed on non‑host reads using MEGAHIT. Contigs longer than 500 base pairs (bp) were analyzed sequentially as follows: first, DIAMOND BLASTx against the NCBI non‑redundant (nr) protein database to identify viral sequences that might be divergent at the nucleotide level but retain protein homology; second, viral sequence prediction using ViralRecall; and finally, classification of predicted viral sequences via BLASTn against a custom viral database. The fourth module addresses read count normalization and positive identification criteria. To account for variations in sequencing depth among samples and enable meaningful comparisons of relative viral abundance across different sample types, read counts were normalized to Reads Per Million (RPM). RPM was calculated using the following formula: RPM = (Number of reads uniquely mapped to a specific virus / Total number of high‑quality non‑host reads in the sample) × 1,000,000. The use of non‑host reads as the denominator (rather than all raw or quality‑controlled reads) avoids artificial inflation or deflation of viral abundance caused by variable host DNA content across different sample types. This normalization approach ensures that RPM values accurately reflect the relative abundance of viral sequences within the microbial fraction of each sample.

A virus was considered positively identified if it met all of the following criteria: normalized read abundance > 2 RPM, a consensus sequence or contig > 150 bp, and nucleotide/amino acid identity and coverage meeting reliable thresholds for the viral taxon. Viral reads with normalized abundance ≤ 2 RPM were classified as “sparse reads” and were not considered confirmed positive detections, although they were still recorded for descriptive purposes in the viral community composition analysis. For viruses meeting these criteria, key metrics including read counts, average sequencing depth, and reference genome coverage were calculated per sample. For taxonomic classification, viral reads and contigs were assigned using a tiered approach. Primary taxonomic assignment was conducted at the family level; for contigs or consensus sequences with sufficient alignment coverage and read depth, species‑level identification was further achieved by BLASTn against the NCBI non‑redundant nucleotide sequences (NT) database, complemented by phylogenetic analysis or genotyping tools as appropriate. For detection signals supported only by sparse reads, classification remained at the family level. Downstream statistical and visualization analyses included BLAST‑based sequence validation against the NCBI database with homologous reference sequences downloaded from GenBank, MAFFT‑based multiple sequence alignment, beta‑diversity analysis (Bray‑Curtis distance) using the vegan package in R (version 4.2), and visualization via Principal Coordinates Analysis (PCoA), hierarchical clustering (UPGMA), and Venn diagrams.

The complete MapV pipeline, including source code, detailed parameter configurations, and implementation documentation, has been previously described in our published methodological studies[18], [19].

For the hyperplex targeted sequencing data, reads were aligned to a database containing the 139 target RNA viral genomes using BWA-MEM (v0.7.17). A detection was considered positive if ≥ 10 uniquely mapped reads covered ≥ 50% of a target genome.

### 2.7 Statistical analysis

Categorical variables were summarized as frequencies and proportions (%). Continuous variables were tested for normality using the Shapiro–Wilk test; those not following a normal distribution were reported as median with interquartile range (IQR). No formal hypothesis testing was conducted for group comparisons, as this was an exploratory case series without an external control group. All reported results are descriptive in nature.

## 3 Results

### 3.1 Sequencing data quality and host depletion

The hyperplex targeted sequencing generated 44.4 million reads (3.30 Gbp), achieving 100% Q30 scores. Metagenomic sequencing produced 1.42 billion paired-end reads (427.36 Gbp), with Q30 values ranging from 90.9% to 96.7%, indicating excellent data quality. Post-host depletion, non-host sequences accounted for 95.7% and 63.9% of total reads in fecal and plasma samples, respectively.

### 3.2 Clinical data analysis

This study enrolled 49 patients (48 adults and one adolescent aged 16 years) with hepatitis of unknown origin, all of whom underwent comprehensive etiological screening. Through detailed questionnaires and clinical record reviews, all participants explicitly denied a history of chronic or excessive alcohol consumption (defined as < 30 g/day for men and < 20 g/day for women), effectively excluding alcohol-induced liver injury. A thorough medication history review and temporal analysis revealed no exposure to prescription drugs, herbal medicines, or dietary supplements known to cause drug-induced liver injury. Occupational exposure assessments confirmed no contact with hepatotoxic agents, including organic solvents, heavy metals, or other known liver toxins. Furthermore, serological testing and clinical evaluations ruled out common etiologies such as autoimmune hepatitis, metabolic liver diseases, and biliary disorders. Regrettably, all 49 patients declined liver biopsy, precluding histopathological confirmation.

The cohort comprised 18 males (36.7%) and 31 females (63.3%). The median age was 53.0 years overall (IQR: 42.0–62.0), 50.5 years in males (IQR: 42.5–60.0), and 53.0 years in females (IQR: 43.0–62.5). The youngest patient was 16 years old; however, all participants were legally adults (≥18 years) at the time of enrollment, with the exception of one 16-year-old patient who was enrolled with parental consent and treated in the adult hepatology department due to transfer from pediatric care. Regarding clinical presentation, 7 patients (14.3%) presented with symptoms including fatigue, nausea, and anorexia, while the remaining 42 patients (85.7%) were asymptomatic and identified through abnormal liver function tests during routine health check-ups or pre-operative screening.

Elevated ALT was observed in 51.0% of patients (25/49), while 46.9% (23/49) had increased AST. Hyperbilirubinemia (total bilirubin) was present at 28.6% (14/49), elevated direct bilirubin in 36.7% (18/49), hypoalbuminemia (albumin) in 40.8% (20/49), raised gamma-glutamyl transferase (GGT) in 49.0% (24/49), and elevated alkaline phosphatase (ALP) in 34.7% (17/49). Hematological abnormalities included leukopenia (white blood cell count < 3.5 × 10^9^/L: 12.2%, 6/49), neutropenia (absolute neutrophil count < 1.8 × 10^9^/L: 12.2%, 6/49), and thrombocytopenia (platelet count < 125 × 10^9^/L: 18.4%, 9/49) (Table 1).

**Table 1:** Baseline demographic and laboratory profiles of 49 patients with HUO at enrollment.

Characteristics	Total (n = 49)	Male (n = 18)	Female (n = 31)
Age, median (years, IQR: 25^th^–75^th^)	53.0 (42.0–62.0)	50.5 (42.5–60.0)	53.0 (43.0–62.5)
ALT >40 U/L	25 (51.0)	10 (55.6)	15 (48.4)
AST >35 U/L	23 (46.9)	9 (50.0)	14 (45.2)
Tbil >18.8 μmol/L	14 (28.6)	6 (33.3)	8 (25.8)
Dbil >6.8 μmol/L	18 (36.7)	7 (38.9)	11 (35.5)
ALB <40 g/L	20 (40.8)	6 (33.3)	14 (45.2)
GGT >60 U/L	24 (49.0)	8 (44.4)	16 (51.6)
ALP >125 U/L	17 (34.7)	6 (33.3)	11 (35.5)
CHE <4,000 U/L	5 (10.2)	2 (11.1)	3 (9.7)
WBC <3.5 × 10^9^/L	6 (12.2)	1 (5.6)	5 (16.1)
NE# <1.8 × 10^9^/L	6 (12.2)	1 (5.6)	5 (16.1)
PLT <125 × 10^9^/L	9 (18.4)	4 (22.2)	5 (16.1)

Data are presented as median (IQR: 25^th^–75^th^) for continuous variables and as n (%) for categorical variables. Data were obtained from blood samples collected at enrollment (baseline). Abbreviations: HUO, hepatitis of unknown origin; IQR, interquartile range; ALT, alanine aminotransferase; U/L, units per liter; AST, aspartate aminotransferase; Tbil, total bilirubin; Dbil, direct bilirubin; ALB, albumin; GGT, gamma-glutamyl transferase; ALP, alkaline phosphatase; CHE, cholinesterase; WBC, white blood cell; NE#, Neutrophil Absolute Count; #, number; PLT, platelet count.

### 3.3 Etiological detection results

In this study, we performed an integrated pathogen screening approach combining highly multiplexed targeted RNA viral sequencing and total RNA metagenomic sequencing on 59 clinical specimens (44 plasma and 15 fecal samples) from 49 patients with hepatitis of unknown etiology. The targeted sequencing achieved exceptional data quality with 100% Q30 scores and an average of 0.75 million reads per sample, enabling sensitive detection of 139 predefined RNA viral targets. Simultaneously, the metagenomic sequencing generated robust datasets averaging 7.24 Gbp per sample with high-quality bases (Q30: 90.9%–96.7%), while effectively depleting host DNA to 4.3% in fecal and 36.1% in plasma samples.

#### 3.3.1 Hyperplex targeted RNA sequencing

Our highly multiplexed targeted sequencing analysis of 139 RNA viruses detected no viral signals in 58/59 samples (98.3%). The only positive case was the identification of HCV in a plasma sample from a 60-year-old male patient (sample p2501). This detection was subsequently confirmed by follow-up RT-PCR and serology, as detailed below. These results indicate that the remaining 58 samples cannot be explained by common RNA viral pathogens covered by this panel.

#### 3.3.2 Metagenomic sequencing

To comprehensively investigate potential pathogens, we performed unbiased metagenomic sequencing on all 59 samples. Viral family-level analysis identified 24 taxonomic groups, including bacteriophages, human pathogens, plant viruses, and unclassified viruses. Given the clinical focus on hepatitis etiology, Table 2 highlights the six viral groups that are known or suspected to infect humans and have potential clinical relevance; the remaining groups were mainly bacteriophages, plant viruses, and unclassified viruses and are not individually presented. However, when applying our strict positive identification criteria (RPM > 2, contig length > 150 bp, and significant sequence identity), no apparently enriched novel or rare viruses with clear pathogenic potential were detected. While some human-infecting viruses like *Anelloviridae* and *Herpesviridae* were identified at the family level (Table 2), most individual virus species within these families were detected at very low read counts (below the RPM > 2 threshold) and were thus considered sparse reads, not confirmed positive detections.

**Table 2:** Summary of selected viral sequencing findings in plasma and fecal samples.

Virus classification	Taxonomic	n (No. of samples with detected viral signals)	Sample type	Identification results
Anellovirus	*Anelloviridae*	26	Plasma	−
Herpesvirus	*Herpesviridae*	11	Fecal sample	−
Circovirus	*Circoviridae*	1	Fecal sample	GoCV
Flavivirus	*Flaviviridae*	1	Plasma	HCV
Orthomyxovirus	*Orthomyxoviridae*	1	Plasma	Unconfirmed influenza A-like signal
Hepatitis virus	*Hepadnaviridae*	1	Plasma	HBV

Abbreviations: GoCV, goose circovirus; HCV, hepatitis C virus; FluA, influenza A virus; HBV, hepatitis B virus; No., number.

Notably, a circovirus-like sequence showing high genomic identity to goose circovirus (GoCV) was detected in one fecal sample (f3961, 6.7%, 1/15) and was not detected in any plasma sample (0.0%, 0/44), leaving its causal relationship with hepatitis unclear. As shown in Table 3, the virus identified in this study (f3961) demonstrated 95.9% sequence identity with the goose circovirus reference strain NC_003054.1. This sequence shares only 31.9% identity with the goose circovirus isolate GD-HK-2022 (GenBank accession PP968832.1) previously reported from Hong Kong, China, indicating that the detected virus belongs to the goose circovirus species and is distinct from previously reported circovirus sequences associated with human hepatitis cases.

**Table 3:** Pairwise nucleotide identity matrix based on BLAST analysis against NCBI reference sequences.

Virus strains	*f3961*	*PP968832.1*	*NC_003054.1*	*NC_038392.1*	*OL456416.1*	*NC_007220.1*	*NC_006232.1*
*f3961*	−	31.9%	95.9%	89.9%	56.6%	34.7%	37.0%
*PP968832.1*	31.9%	−	32.1%	32.4%	36.1%	30.1%	30.7%
*NC_003054.1*	95.9%	32.1%	−	93.0%	57.8%	35.0%	37.9%
*NC_038392.1*	89.9%	32.4%	93.0%	−	58.7%	34.8%	38.6%
*OL456416.1*	56.6%	36.1%	57.8%	58.7%	−	34.9%	36.6%
*NC_007220.1*	34.7%	30.1%	35.0%	34.8%	34.9%	−	38.9%
*NC_006232.1*	37.0%	30.7%	37.9%	38.6%	34.9%	38.9%	−

Values represent the proportion of identical nucleotide sites between two sequences. Sequence identities were determined using BLASTn against the NCBI non-redundant nucleotide (NT) database.

Abbreviations: –, not applicable (self-comparison). GenBank accession numbers corresponding to strain abbreviations: f3961, circovirus strain f3961 (this study); PP968832.1, human-derived circovirus; OL456416.1, goose circovirus strain GD-01–2021 (China); NC_003054.1, goose circovirus reference genome; NC_007220.1, goose circovirus strain D98-545; NC_038392.1, goose circovirus strain GDC; NC_006232.1, goose circovirus strain AH.

Metagenomic sequencing also identified HBV sequences in one plasma sample (patient p9675), with 1,005 reads, 76.5% genome coverage (2,658 bp), and an RPM of 7.43. The assembled sequence showed 94.6% nucleotide identity to the HBV reference genome. However, the patient tested negative for HBsAg and HBV DNA by standard serological and PCR assays at enrollment, and no other markers of active HBV replication were present. This finding may represent occult HBV infection (OBI), although a definitive causal relationship with the patient's hepatitis could not be established based on the available data.

For patient p2501, in whom targeted sequencing had identified HCV, the metagenomic sequencing assembled a near-full-length HCV genome (9,070 bp, 94.5% genome coverage) with 92.4% nucleotide identity to the HCV genotype 1b reference genome L02836. At enrollment, the initial RT-PCR (lower limit of detection: 15 IU/mL) and anti-HCV serological testing performed on a plasma sample collected at enrollment were both negative, which initially led to the patient's classification as HUO. The metagenomic sequencing was performed on a separate aliquot of the same blood draw. Following the metagenomic detection, a follow-up plasma sample was obtained 14 days after enrollment to verify the finding. Repeat RT-PCR testing on this follow-up sample confirmed HCV RNA positivity with a viral load of 4.2 × 105 IU/mL, and anti-HCV antibody seroconversion was also documented by chemiluminescent immunoassay (CLIA). These follow-up results confirmed that patient p2501 had been in the seronegative window period of acute HCV infection at the time of enrollment, with the initial negative RT-PCR attributable to viral RNA levels below the assay's limit of detection.

Additionally, an unconfirmed low-level influenza A-like signal was detected in a single plasma sample (p6289). The signal was characterized by low read counts (20 reads for segment 4, 16 reads for segment 6), incomplete genome coverage, and RPM values below the > 2 confirmation threshold (RPM 1.26 and 1.01 for segments 4 and 6, respectively) (Table 4). Attempts to confirm this signal by influenza A virus-specific RT-qPCR on the same plasma sample were unsuccessful. Detailed clinical and laboratory characteristics of this case are presented in Table 5. Given that this signal did not meet our predefined criteria for confirmed positive detection, it is reported here as an unconfirmed below-threshold sequencing finding rather than a confirmed influenza A virus infection.

**Table 4:** Key metrics of candidate viruses from metagenomic sequencing.

Virus	ID	Reads	Contig length (bp)	RPM	Genome coverage (%)	Nucleotide identity (%)	Reference sequence (BLAST top hit)
GoCV	f3961	55	1,821	3.82	96.5	95.9	NC_003054.1
HCV	p2501	692	9,070	5.14	94.5	92.4	L02836 (AB049008)
HBV	p9675	1,005	2,658	7.43	76.5	94.6	NC003977 (DQ315776)
FluA	p6289s4	20	891	1.26	51.9	97.5	NC007362 (LC765309)
FluA	p6289s6	16	654	1.01	44.1	99.6	NC007361 (LC762442)

Abbreviations: ID, identifier; RPM, reads per million non-host reads; GoCV, goose circovirus; HCV, hepatitis C virus; HBV, hepatitis B virus; FluA, unconfirmed low-level influenza A-like sequencing signal. “Genome coverage (%)” refers to the percentage of the reference genome covered by the assembled contig or mapped reads. “Nucleotide identity (%)” was determined by BLASTn against the indicated reference sequence or BLAST top hit.

**Table 5:** Clinical and virological characteristics of cases with notable viral sequencing signals.

Characteristics	HCV-positive (sample p2501)	HBV-positive (sample p9675)	Influenza A–like signal (sample p6289)
Age/sex	60 years, male	55 years, female	65 years, female
Main symptoms	Not documented	Not documented	Fatigue, anorexia, nausea
ALT (U/L)	9.1	72.6	246.6
AST (U/L)	15.3	37.5	187.4
Peak ALT (U/L)	1,040.9	Not determined	Not determined
Peak AST (U/L)	972.6	Not determined	Not determined
Date of peak value	3 weeks before enrollment	−	−
Total bilirubin (μmol/L)	18.2	10.8	24.4
Virus detected	HCV genotype 1b	HBV	Unconfirmed influenza A-like signal
Sequencing reads	692	1,005	36 (total; 20 reads for segment 4, 16 reads for segment 6)
Genome coverage (by all reads)	94.5% (9,070 bp)	76.5% (2,658 bp)	51.9% (segment 4), 44.1% (segment 6)
RPM	5.14	7.43	1.26 (segment 4), 1.01 (segment 6)
Virological note	92.4% identity to HCV 1b reference genome	94.6% identity to HBV reference genome	Low viral load, incomplete genome coverage; detection below RPM > 2 threshold

Abbreviations: HCV, hepatitis C virus; ALT, alanine aminotransferase; U/L, units per liter; AST, aspartate aminotransferase; bp, base pair; RPM, reads per million non-host reads.

Comparative analysis of the overall viral community structure revealed apparent compositional differences between plasma and fecal samples. Heatmap and clustering analyses demonstrated distinct segregation of viral profiles, with the two sample types forming separate clusters (Fig. 1). Venn diagram analysis further showed that plasma and feces shared only 5 viral categories (primarily bacteriophages and unclassified viruses), while feces contained 15 unique viral types and plasma harbored 4 exclusive types—all known human pathogens (Fig. S1). Notably, in the 9 patients with paired plasma–fecal samples, we observed virtually no viral overlap between sample types, suggesting highly compartmentalized viral communities without evidence of cross-niche transmission within this cohort.

**Fig. 1:**
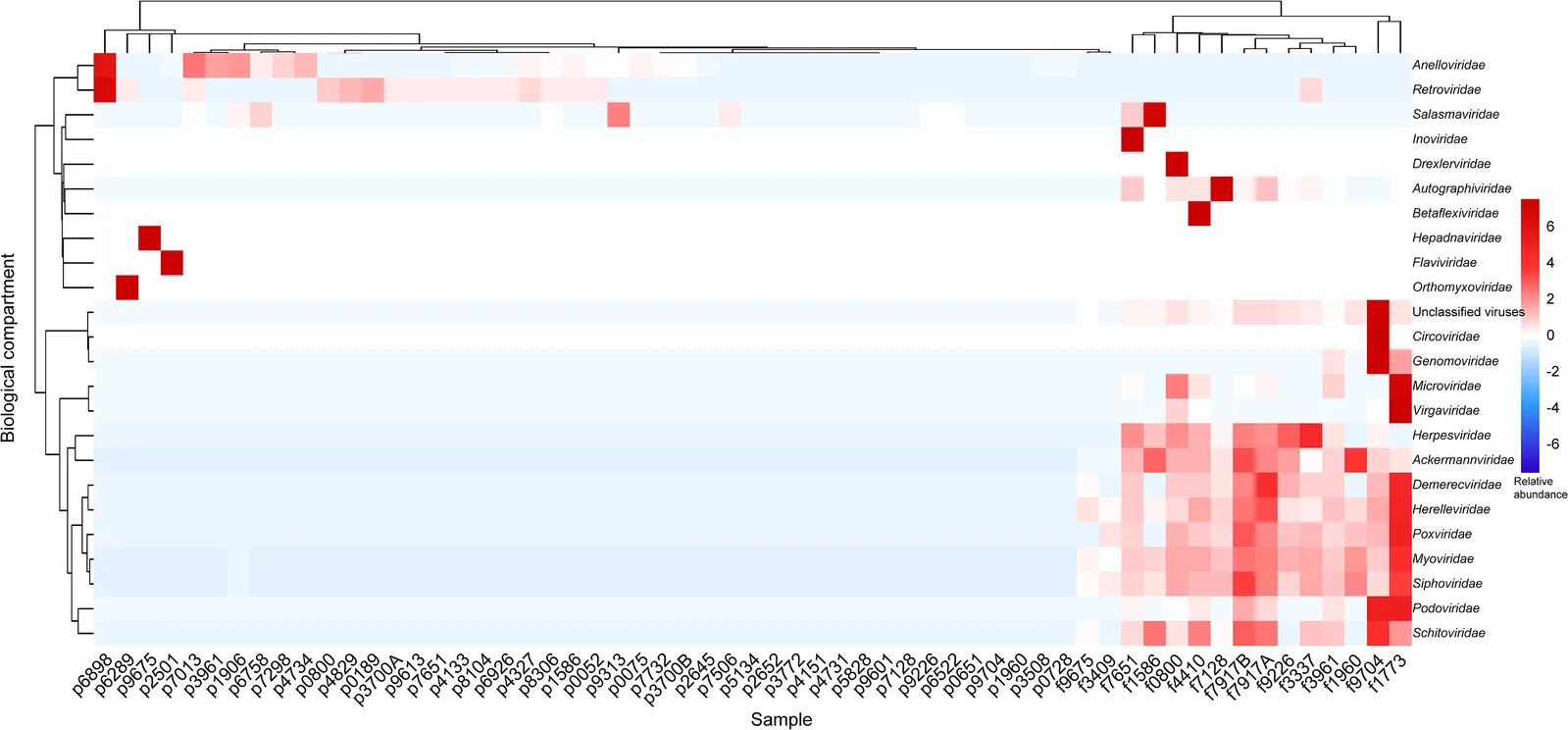
Heatmap and clustering of samples based on viral family relative abundance. Sample identification numbers (IDs) correspond to the last four digits of the registration number in the specimen registry, with “f” denoting fecal samples and “p” indicating plasma samples. The heatmap displays the relative abundance of detected viral families across all 59 samples (44 plasma ‘p’, 15 fecal ‘f’). Samples were grouped using hierarchical clustering based on their viral composition. As shown, plasma and fecal samples exhibited apparent differences in viral community structure, forming two major clusters, indicating niche specificity of viral communities in different biological compartments. This is an unsupervised analysis based on data from all enrolled cases.

## 4 Discussion

This study employed metagenomic approaches to screen for pathogens and characterize the virome in a cohort of 49 patients with HUO (predominantly adults, aged 16–74 years). As an exploratory case series, its primary objective was to describe the virome and explore potential pathogen profiles within this specific clinical cohort. Therefore, this study was not designed with an external healthy control group for traditional case-control comparisons aimed at establishing disease associations. Consequently, we refrain from making definitive claims regarding statistical associations between specific viruses and disease state. Instead, our analysis focused on internal comparisons between plasma and fecal compartments. The hierarchical clustering heatmap in Fig. 1 visually represents this community structure divergence, depicting the natural grouping of all samples based on viral composition, which suggests niche-specific partitioning rather than disease-associated enrichment. Together, these findings suggest that no single virus was universally present across this HUO cohort, supporting an etiologically heterogeneous landscape. A goose circovirus-like sequence was detected in one fecal specimen and showed high sequence identity to the goose circovirus reference strain NC_003054.1, and its key mapping metrics are summarized in Table 4. However, as there is no evidence that goose circovirus can infect humans or cause hepatitis, its detection likely reflects environmental exposure or dietary contamination rather than true infection [20].

In recent years, the application of metagenomic technologies has made the pathogen exploration of hepatitis of unknown origin a research hotspot. During the 2022 global outbreak of acute hepatitis in children, multiple studies using metagenomics proposed hypotheses involving adenovirus, adeno-associated virus type 2, and host immune interactions, yet failed to confirm a dominant role for any single pathogen [21]. Our study extends this focus to patient populations with hepatitis of unknown origin, including adults, with results similarly pointing toward etiological heterogeneity and multifactorial involvement. Compared to pediatric cases, the pathogen spectrum in this predominantly adult cohort may be more complex, potentially involving mechanisms such as chronic infections, immune memory, and environmental exposures [22].

The dual sequencing strategy employed in this study provided both depth for known pathogen detection and breadth for unbiased microbial discovery, establishing a high-quality data foundation for comprehensive microbial analysis. The optimized host DNA removal protocol significantly enhanced pathogen detection sensitivity, particularly in plasma samples where human genomic content typically predominates, ensuring reliable identification of potential etiological agents in this challenging clinical cohort.

A noteworthy finding was the detection of known hepatitis viruses (HCV and HBV) by metagenomics in patients initially classified as HUO based on standard serological testing. For patient p2501, the assembled HCV contig exhibited 94.5% genome coverage (9,070 bp of the 9.6 kb reference genome) with 92.4% nucleotide identity to the HCV genotype 1b reference genome GenBank: L02836, and 90.9% identity to the closely related strain AB049008 by BLAST analysis. The identification of a near-full-length HCV genotype 1b genome (9,070 bp, 94.5% genome coverage) in patient p2501 is likely a case of seronegative window-period infection. At the time of enrollment, the patient's serological tests for anti-HCV antibodies and HCV RNA (via standard RT-PCR, limit of detection 15 IU/mL) were both negative, leading to their classification as HUO. However, metagenomic sequencing subsequently identified a nearly complete HCV genome on a separate aliquot of the same blood draw. This finding was confirmed by follow-up RT-PCR and serologic testing performed 14 days after enrollment, which demonstrated HCV RNA positivity with a viral load of 4.2 × 105 IU/mL and anti-HCV antibody seroconversion, ultimately leading to case reclassification. The time interval between the initial negative RT-PCR and subsequent positive detection was approximately 14 days, consistent with the serological window period of acute HCV infection. This observation highlights the potential advantage of unbiased nucleic acid detection methods in identifying pathogens that may fall below the detection threshold of conventional serological or targeted PCR assays, particularly during the early seronegative phase of infection [23]. The isolated, low-level HBV sequences (1,005 reads, 76.5% genome coverage, RPM 7.43) detected in patient p9675 may represent occult HBV infection (OBI), defined as the presence of HBV DNA in the liver or blood of individuals testing negative for hepatitis B surface antigen (HBsAg) [24]. OBI has been well-documented in patients with cryptogenic liver disease and may contribute to liver inflammation through direct cytopathic effects or immune-mediated mechanisms, even at very low viral loads[25], [26]. However, given the low viral load, the isolated nature of this detection, and the absence of other serological markers of active HBV replication in our cohort, a clear causal association with the current hepatitis activity could not be established. These observations collectively highlight the complementary value of metagenomic sequencing in the diagnostic workup of hepatitis, especially when serological results are inconclusive or during the early stages of infection. Furthermore, they emphasize that the diagnosis of HUO should be viewed as a working diagnosis based on available clinical testing, which may be refined or reclassified as more sensitive detection technologies become available. The integration of metagenomics into routine diagnostic algorithms could potentially reduce the proportion of HUO cases and facilitate earlier, more precise therapeutic interventions.

Our virome analysis revealed distinct compartmentalization of viral communities between plasma and fecal samples. The two sample types shared only a limited number of bacteriophages and unclassified viruses, while known human pathogenic viruses (particularly *Anelloviridae* and *Flaviviridae*) were predominantly detected in plasma. These findings are consistent with recent microbiome studies [27], [28], [29], demonstrating the remarkable niche specificity of viral communities in different biological compartments. Notably, in the nine paired plasma–fecal samples, we observed no co-detection of identical viral species, which may suggest restricted viral cross-compartment dissemination or simply reflect the limited sample size. However, given the small number of paired samples (n = 9), this observation should be interpreted with caution and requires validation in larger cohorts with matched specimens. This compartmentalization likely reflects either host immune surveillance efficiency or intrinsic viral tropism, and underscores the importance of multi-sample profiling in comprehensive pathogen screening to avoid potential detection bias. Importantly, while the nucleic acid extraction protocols employed for plasma and fecal samples were methodologically aligned in terms of total nucleic acid extraction (encompassing both DNA and RNA), they utilized different matrix-specific kits (Table S2). This methodological distinction may introduce potential biases in the comparative recovery of certain virus types across the two compartments. The observed viral community divergence between plasma and fecal compartments should therefore be interpreted with this technical consideration in mind, and the possibility that some differences may be influenced by extraction efficiency cannot be entirely excluded. The shared detection of certain virus families (including both DNA and RNA viruses) across both compartments, alongside the unique viruses identified in each niche, suggests genuine biological compartmentalization, but further studies using harmonized extraction protocols would be valuable to confirm these findings.

In a 2024 study from Hong Kong, China, human circovirus viremia and sequences were identified in eight hepatitis patients [30]. Specifically, our sequence (f3961) exhibited 95.9% identity with the goose circovirus reference strain NC_003054.1, but only 56.6% identity with the Guangdong goose circovirus strain OL456416 and 31.9% identity with the human-derived circovirus strain PP968832.1 from Hong Kong, China. However, its detection showed no correlation with disease status, and current evidence does not support its ability to infect humans or cause hepatitis [31]. Members of the *Circoviridae* family primarily infect birds and mammals, with extremely rare reports of human infection mostly limited to immunocompromised individuals [32].

Furthermore, in this metagenomics-based etiological investigation of hepatitis of unknown origin, we detected an unconfirmed low-level sequencing signal resembling influenza A virus in a plasma sample from one patient. Although this was an isolated case with very low read counts below our predefined RPM > 2 confirmation threshold, recent research developments suggest the need to consider the potential role of respiratory viruses in liver injury. As a common respiratory pathogen, influenza virus can indeed cause hepatic damage, though this is not typically present as a primary clinical manifestation. Currently, there are limited reports on liver injury caused by influenza virus, and its specific mechanisms remain unclear. The detection of an influenza A-like sequencing signal in this study occurred after excluding classic hepatotropic viruses. Therefore, while the single low-titer signal in our study is insufficient to recommend routine screening for influenza A virus in patients with HUO, it highlights that in patients with concurrent or recent significant respiratory symptoms, PCR testing for respiratory viruses, including influenza, may be clinically justified to rule out viremia-associated liver injury as a differential diagnosis. However, given that only a single low-signal sample of influenza A-like sequences was detected in this study, and the signal did not meet our strict positive identification criteria, establishing a direct causal relationship with hepatitis remains challenging. The finding most likely represents incidental sequencing signal, transient viremia, or laboratory contamination, but these interpretations remain speculative given the below-threshold nature of the detection.

Several limitations of this study should be acknowledged. First, the lack of liver tissue samples from all patients precludes histopathological confirmation and limits the investigation of intrahepatic pathogen localization, immune cell infiltration profiles, and direct mechanisms of tissue injury. Second, while influenza A virus genomic sequences were detected in one plasma sample with low viral load, this isolated, low-level detection—which could represent incidental carriage or co-infection—is currently insufficient to recommend routine screening for influenza virus as an etiological agent in patients with HUO. The significance of this finding lies rather in suggesting that PCR-based testing for respiratory viruses, including influenza, could be considered in hepatitis patients with concurrent prominent respiratory symptoms; however, any causal relationship requires validation in larger cohorts. Third, as an exploratory case series, this study was not designed with an external healthy control group for traditional case-control comparisons. While this limits our ability to definitively associate a detected virus with the disease, our internal comparisons between plasma and fecal compartments and the lack of any single virus being universally enriched across all cases provide valuable insights into the etiological heterogeneity of HUO. We acknowledge this as a key limitation and emphasize the need for larger, controlled cohort studies to validate our findings. Fourth, the relatively small sample size (n = 49), particularly for paired plasma-fecal samples (n = 9) and fecal samples alone (n = 15), may limit the power to detect rare or low-abundance pathogens. Fifth, the availability of peak transaminase values varied across patients: for those who presented directly to our center, complete laboratory data were available; however, for referred patients, peak values from referring hospital records were not always obtainable due to incomplete documentation or loss of follow-up data. For the HCV-positive patient (p2501), the peak ALT and AST levels (1040.9 U/L and 972.6 U/L, respectively) were documented in the referring records (occurring 3 weeks prior to enrollment), as shown in Table 5; for other patients without available peak values, we relied on the enrollment laboratory findings that still met the inclusion criteria combined with persistent clinical symptoms to confirm active hepatitis. This reflects the practical challenges of multicenter data collection and highlights the need for prospective studies with harmonized laboratory data capture across referring and enrolling centers. Larger multicenter cohort studies are warranted to confirm our findings.

## Ethics statement

Written informed consent to take part in the study and to publish the article has been obtained from all participants or their legal representatives. The privacy rights of participants have been observed.

All procedures performed in this study involving human participants followed the ethical standards of the Ethics Committee of Beijing Ditan Hospital, Capital Medical University (DTEC-KY2023-046-01) and the 1964 Helsinki Declaration and its later amendments or comparable ethical standards. The authors have obtained written informed consent from all participants prior to their inclusion in the study, after providing detailed explanations of the study objectives, procedures, potential risks, and benefits. For the one adolescent participant aged 16 years, written informed consent was obtained from both the participant and their parent/legal guardian, in accordance with applicable regulations governing minors' participation in clinical research. To ensure privacy, all collected data were anonymized and used exclusively for scientific research purposes. The results of this clinical trial and any associated work have been posted in a registry. This clinical trial was registered with number NCT06653140 (Clinicaltrials).

## Supplementary data

Supplementary Data 1 Patient and sample summary, nucleic acid extraction methods, targeted RNA viral panel, and virome distribution in plasma and fecal samples from patients with hepatitis of unknown origin.
