# Supplementary material for: Metagenomic study in a predominantly adult cohort with hepatitis of unknown etiology
Source: Biosaf Health. 2026 Sep 18;8(5):359–67. doi: 10.1016/j.bsheal.2026.09.003 (PMC13729065; doi:10.1016/j.bsheal.2026.09.003)
Supplement: Supplementary file 1 [file mmc1.docx]

**Table S1 Patient and sample summary**

| Category | Number |
| --- | --- |
| Total enrolled patients | 49 |
| Age range (years) | 16–74 |
| Sex (male/female) | 18 / 31 |
| Blood only | 34 |
| Fecal only | 6 |
| Matched blood-fecal pairs | 9 |
| Plasma samples | 44 (from 43 patients; 1 patient provided 2 time-point samples) |
| Fecal samples | 15 (from 14 patients; 1 patient provided paired acute-recovery samples) |
| Specimens subjected to sequencing | 59 (44 plasma + 15 fecal) |

**Table S2 Comparison of nucleic acid extraction methods for plasma and fecal samples**

| Sample type | Extraction strategy | Extraction kit |
| --- | --- | --- |
| Plasma | Magnetic bead-based method | Ex-DNA/RNA nucleic acid extraction rapid kit (Bioperfectus, China) |
| Feces | Magnetic bead-based method | MagaBio stool pathogen DNA/RNA purification kit |

**Abbreviations:** DNA, deoxyribonucleic acid; RNA, ribonucleic acid.

Fig. S1 Distribution of virus families across plasma and fecal samples.

Venn diagram illustrates the overlap of virus families detected in plasma (n=44) and fecal (n=15) specimens. Both sample types underwent total nucleic acid extraction (DNA and RNA), enabling unbiased cross-compartment virome comparison. Numbers within the diagram indicate the number of viral families identified in each compartment and their intersection. The shared families primarily comprised bacteriophages and unclassified viruses, while known human pathogenic viruses were predominantly detected in plasma.

**Table S3 Complete list of 139 RNA viral species targeted by the hyperplex targeted sequencing panel.**

| panel | Latin name | English Name |
| --- | --- | --- |
| RNAvirus | Lassa mammarenavirus | Lassa fever virus |
| RNAvirus | Severe acute respiratory syndrome-related coronavirus | Severe acute respiratory syndrome coronavirus (SARS-CoV) |
| RNAvirus | Betacoronavirus 1 | Coronavirus (low pathogenicity to human) |
| RNAvirus | Alongshan virus |  |
| RNAvirus | Zaire ebolavirus | Ebola virus |
| RNAvirus | Echovirus E1 | ECHO virus |
| RNAvirus | Echovirus E11 | ECHO virus |
| RNAvirus | Echovirus E12 | ECHO virus |
| RNAvirus | Echovirus E14 | ECHO virus |
| RNAvirus | Echovirus E16 | ECHO virus |
| RNAvirus | Echovirus E18 | ECHO virus |
| RNAvirus | Echovirus E19 | ECHO virus |
| RNAvirus | Echovirus E2 | ECHO virus |
| RNAvirus | Echovirus E20 | ECHO virus |
| RNAvirus | Echovirus E25 | ECHO virus |
| RNAvirus | Echovirus E27 | ECHO virus |
| RNAvirus | Echovirus E3 | ECHO virus |
| RNAvirus | Echovirus E30 | ECHO virus |
| RNAvirus | Echovirus E4 | ECHO virus |
| RNAvirus | Echovirus E5 | ECHO virus |
| RNAvirus | Echovirus E6 | ECHO virus |
| RNAvirus | Echovirus E7 | ECHO virus |
| RNAvirus | Echovirus E9 | ECHO virus |
| RNAvirus | Aichivirus A | ECHO virus |
| RNAvirus | Rhinovirus A | Rhinovirus |
| RNAvirus | Rhinovirus B | Rhinovirus |
| RNAvirus | Rhinovirus C | Rhinovirus |
| RNAvirus | Hepacivirus C | Hepatitis C virus |
| RNAvirus | Influenza C virus | Influenza virus |
| RNAvirus | Mamastrovirus | Astrovirus |
| RNAvirus | Mamastrovirus 1 | Astrovirus |
| RNAvirus | Mamastrovirus 3 | Astrovirus |
| RNAvirus | Mamastrovirus 4 | Astrovirus |
| RNAvirus | Enterovirus D68 | Enterovirus |
| RNAvirus | Enterovirus A | Enterovirus A-71 |
| RNAvirus | Enterovirus A71 | Enterovirus A-71 |
| RNAvirus | Enterovirus B | Enterovirus A-71 |
| RNAvirus | Enterovirus C | Enterovirus A-71 |
| RNAvirus | Enterovirus D | Enterovirus A-71 |
| RNAvirus | Enterovirus D70 | Enterovirus A-71 |
| RNAvirus | Dengue virus | Dengue virus |
| RNAvirus | Hepatitis delta virus | Hepatitis D virus |
| RNAvirus | Eastern equine encephalitis virus | Eastern equine encephalitis virus |
| RNAvirus | Omsk hemorrhagic fever virus | Omsk hemorrhagic fever virus |
| RNAvirus | Rubivirus rubellae | Rubivirus (Rubella) |
| RNAvirus | Pegivirus A |  |
| RNAvirus | Pegivirus C |  |
| RNAvirus | Hendra henipavirus | Hendra virus |
| RNAvirus | Yellow fever virus | Yellow fever virus, (vaccine strain, 17D) |
| RNAvirus | Chikungunya virus | Chikungunya virus |
| RNAvirus | Hepatovirus A | Hepatitis A virus |
| RNAvirus | Influenza A virus | Influenza virus |
| RNAvirus | Influenza A virus (H10N8) | Influenza virus |
| RNAvirus | Influenza A virus (H1N1) | Influenza virus |
| RNAvirus | Influenza A virus (H2N2) | Influenza virus |
| RNAvirus | Influenza A virus (H3N2) | Influenza virus |
| RNAvirus | Influenza A virus (H5N1) | High pathogenic avian influenza virus |
| RNAvirus | Influenza A virus (H5N6) | High pathogenic avian influenza virus |
| RNAvirus | Influenza A virus (H7N9) | High pathogenic avian influenza virus |
| RNAvirus | Influenza A virus (H9N2) | High pathogenic avian influenza virus |
| RNAvirus | Coxsackievirus A1 | Coxsakie virus |
| RNAvirus | Coxsackievirus A10 | Coxsakie virus |
| RNAvirus | Coxsackievirus A12 | Coxsakie virus |
| RNAvirus | Coxsackievirus A13 | Coxsakie virus |
| RNAvirus | Coxsackievirus A16 | Coxsakie virus |
| RNAvirus | Coxsackievirus A2 | Coxsakie virus |
| RNAvirus | Coxsackievirus A21 | Coxsakie virus |
| RNAvirus | Coxsackievirus A22 | Coxsakie virus |
| RNAvirus | Coxsackievirus A24 | Coxsakie virus |
| RNAvirus | Coxsackievirus A4 | Coxsakie virus |
| RNAvirus | Coxsackievirus A5 | Coxsakie virus |
| RNAvirus | Coxsackievirus A6 | Coxsakie virus |
| RNAvirus | Coxsackievirus A7 | Coxsakie virus |
| RNAvirus | Coxsackievirus A8 | Coxsakie virus |
| RNAvirus | Coxsackievirus A9 | Coxsakie virus |
| RNAvirus | Coxsackievirus B1 | Coxsakie virus |
| RNAvirus | Coxsackievirus B2 | Coxsakie virus |
| RNAvirus | Coxsackievirus B3 | Coxsakie virus |
| RNAvirus | Coxsackievirus B4 | Coxsakie virus |
| RNAvirus | Coxsackievirus B5 | Coxsakie virus |
| RNAvirus | Coxsackievirus B6 | Coxsakie virus |
| RNAvirus | Crimean-Congo hemorrhagic fever orthonairovirus | Crimean-Congo hemorrhagic fever virus (Xinjiang hemorrhagic fever virus) |
| RNAvirus | Rabies lyssavirus | Rabies virus (street virus) |
| RNAvirus | Rift Valley fever phlebovirus | Rift valley fever virus |
| RNAvirus | Primate T-lymphotropic virus 1 | Human T- lymphotropic virus (HTLV) |
| RNAvirus | Primate T-lymphotropic virus 2 | Human T- lymphotropic virus (HTLV) |
| RNAvirus | Primate T-lymphotropic virus 3 | Human T- lymphotropic virus (HTLV) |
| RNAvirus | Mumps orthorubulavirus | Mumps virus |
| RNAvirus | Rotavirus A | Rotavirus |
| RNAvirus | Rotavirus B | Rotavirus |
| RNAvirus | Rotavirus C | Rotavirus |
| RNAvirus | Ross River virus | Ross river virus |
| RNAvirus | Measles morbillivirus | Measles virus |
| RNAvirus | Marburg marburgvirus | Marburg virus |
| RNAvirus | Machupo mammarenavirus | Machupo virus |
| RNAvirus | Nipah henipavirus | Nipah virus |
| RNAvirus | Norwalk virus | Norovirus |
| RNAvirus | Tick-borne encephalitis virus | Tick-borne encephalitis virus |
| RNAvirus | Human coronavirus 229E | Coronavirus (low pathogenicity to human) |
| RNAvirus | Human coronavirus HKU1 | Coronavirus (low pathogenicity to human) |
| RNAvirus | Human coronavirus NL63 | Coronavirus (low pathogenicity to human) |
| RNAvirus | Human coronavirus OC43 | Coronavirus (low pathogenicity to human) |
| RNAvirus | Human respirovirus 1 |  |
| RNAvirus | Human respirovirus 3 |  |
| RNAvirus | Human respiratory syncytial virus A | Respiratory syncytial virus |
| RNAvirus | Human respiratory syncytial virus B | Respiratory syncytial virus |
| RNAvirus | Human poliovirus 1 | Polio virus |
| RNAvirus | Human immunodeficiency virus 1 | Human immunodeficiency virus (HIV) (Type 1 and 2 virus) |
| RNAvirus | Human immunodeficiency virus 2 | Human immunodeficiency virus (HIV) (Type 1 and 2 virus) |
| RNAvirus | Human metapneumovirus | Metapneumovirus |
| RNAvirus | Human orthorubulavirus 2 | Mumps virus |
| RNAvirus | Human orthorubulavirus 4 | Mumps virus |
| RNAvirus | Saint Louis encephalitis virus | St.Louis encephalitis virus |
| RNAvirus | Seoul orthohantavirus | Hantaviruses causing pulmonary syndrome |
| RNAvirus | Parechovirus A | Parechovirus |
| RNAvirus | Parechovirus B | Parechovirus |
| RNAvirus | Louping ill virus | Louping ill virus |
| RNAvirus | Venezuelan equine encephalitis virus | Venezuelan equine encephalitis virus |
| RNAvirus | Orthohepevirus A | Hepatitis E virus |
| RNAvirus | Western equine encephalitis virus | Western equine encephalitis virus |
| RNAvirus | West Nile virus | West Nile virus |
| RNAvirus | SFTS phlebovirus | Dabie bandavirus (SFTS Virus) |
| RNAvirus | SARS-CoV-2 | Severe acute respiratory syndrome coronavirus 2, (SARS-CoV-2) |
| RNAvirus | SARS-CoV-2(Alpha) | Severe acute respiratory syndrome coronavirus 2, (SARS-CoV-2) |
| RNAvirus | SARS-CoV-2(Beta) | Severe acute respiratory syndrome coronavirus 2, (SARS-CoV-2) |
| RNAvirus | SARS-CoV-2(Delta) | Severe acute respiratory syndrome coronavirus 2, (SARS-CoV-2) |
| RNAvirus | SARS-CoV-2(Gamma) | Severe acute respiratory syndrome coronavirus 2, (SARS-CoV-2) |
| RNAvirus | SARS-CoV-2(Omicron BA.5) | Severe acute respiratory syndrome coronavirus 2, (SARS-CoV-2) |
| RNAvirus | SARS-CoV-2(Omicron BF.7) | Severe acute respiratory syndrome coronavirus 2, (SARS-CoV-2) |
| RNAvirus | SARS-CoV-2(Omicron BQ.1.1) | Severe acute respiratory syndrome coronavirus 2, (SARS-CoV-2) |
| RNAvirus | SARS-CoV-2(Omicron XBB.1) | Severe acute respiratory syndrome coronavirus 2, (SARS-CoV-2) |
| RNAvirus | SARS-CoV-2(Omicron) | Severe acute respiratory syndrome coronavirus 2, (SARS-CoV-2) |
| RNAvirus | Influenza B virus | Influenza virus |
| RNAvirus | Japanese encephalitis virus | Japanese encephalitis virus |
| RNAvirus | Sapporo virus | Sapovirus |
| RNAvirus | Zika virus | Zika Virus |
| RNAvirus | Gibbon ape leukemia virus |  |
| RNAvirus | Hantaan orthohantavirus | Hantaviruses causing hemorrhagic fever with renal syndrome |
| RNAvirus | Middle East respiratory syndrome-related coronavirus | Middle East Respiratory Syndrome coronavirus (MERS-CoV) |
